# Advancing Photodynamic Cancer Therapy with Smart Light-Responsive Lipid and Polymeric Nanocarriers: Evidence from a Meta-Analysis of Efficacy and Pharmacokinetics

**DOI:** 10.3390/ph18121796

**Published:** 2025-11-25

**Authors:** Ahmed M. Agiba, Rabab A. El-Gazar, Mohamed A. Mekkawy, Nihal Elsayyad, Hala N. ElShagea, Patricia Segura-Medina, Raghda Rabe Hamed

**Affiliations:** 1 School of Engineering and Science, Tecnológico de Monterrey, Monterrey 64849, Mexico; 2 Department of Clinical Pharmacy, Faculty of Pharmacy, October 6 University, Giza 12585, Egypt; rababahmed@o6u.edu.eg; 3 Health Services Sector, Ministry of Interior, New Cairo 1, Cairo 11835, Egypt; m_mekkawy@yahoo.com; 4 Department of Pharmaceutics and Industrial Pharmacy, Faculty of Pharmacy, October for Modern Sciences and Arts University, Giza 12451, Egypt; nmahdy@msa.edu.eg; 5 Department of Pharmaceutics and Industrial Pharmacy, Faculty of Pharmacy, Ahram Canadian University, Giza 12451, Egypt; hala.nehad@acu.edu.eg; 6 Departamento de Investigación en Hiperreactividad Bronquial, Instituto Nacional de Enfermedades Respiratorias Ismael Cosío Villegas, Calzada de Tlalpan 4502, Mexico City 14080, Mexico; 7 Escuela de Medicina y Ciencias de la Salud, Tecnológico de Monterrey, Mexico City 14380, Mexico; 8 Department of Industrial Pharmacy, College of Pharmaceutical Sciences and Drug Manufacturing, Misr University for Science and Technology (MUST), 6th of October City 12566, Giza, Egypt; raghda.hamid@must.edu.eg

**Keywords:** meta-analysis, light-responsive, lipid and polymeric nanocarriers, pharmacokinetics, bioavailability, controlled drug release, targeted anticancer drug delivery

## Abstract

**Background/Objectives**: Conventional anticancer drugs often exhibit limited solubility and bioavailability due to unfavorable physicochemical properties and inherent physiological barriers. To overcome these persistent challenges, nanocarriers have been developed to enhance drug bioavailability and therapeutic efficacy. Among these, lipid and polymeric nanocarriers (LP-NCs) have emerged as particularly promising candidates for anticancer drug delivery. These systems can be engineered for targeted delivery and tailored to respond to specific stimuli, thereby enhancing their therapeutic potential. A notable advancement in this field is the development of smart light-responsive LP-NCs, which demonstrate superior performance over conventional nanocarriers by enabling controlled drug release in response to external light stimuli. **Methods**: This study presents a meta-analysis based on a curated selection of publications from multiple scientific databases and literature sources. The objective was to evaluate whether light-responsive LP-NCs offer superior anticancer drug bioavailability and therapeutic efficacy compared with their conventional counterparts. The primary outcome measure was the pharmacokinetic parameter area under the curve (AUC), derived from *in vivo* animal studies. **Results**: The analysis revealed a significant increase in AUC for light-responsive LP-NCs, indicating improved drug bioavailability and prolonged systemic exposure. **Conclusions**: These findings highlight the potential of LP-NCs as a promising strategy for enhancing targeted anticancer drug delivery. This approach could pave the way for more effective therapeutic interventions and warrants further investigation in future research and clinical trials.

## 1. Introduction

In recent years, global cancer incidence and mortality rates have risen dramatically, with cancer now accounting for one out of every six deaths and ranking as the second highest cause of death worldwide [1]. The main treatment options currently available for cancer are surgery, radiation therapy, chemotherapy, and immunotherapy [2]. While both surgery and radiation therapy have several distinct advantages, they can also cause serious adverse side effects. For example, patients with prostate cancer may experience incontinence and severe gastrointestinal complications following treatment [3], while female patients undergoing hormone therapy may also face substantial side effects, such as hot flashes, osteoporosis, and an increased risk of cardiovascular disease [4]. In addition, chemotherapy and radiotherapy, which remain cornerstones of cancer treatment, are frequently associated with systemic toxicities, including nausea, fatigue, immunosuppression, and damage to specific organs. These adverse side effects can significantly affect patients’ quality of life and frequently restrict treatment adherence or dosing, ultimately diminishing therapeutic outcomes. Chemotherapeutic drugs are highly effective in cancer treatment; however, their clinical use is often limited by persistent challenges, including the invasive nature of solid tumors, poor aqueous solubility and low bioavailability, short systemic circulation, multidrug resistance, nonspecific targeting, rapid metabolism, and both systemic and local off-target side effects [5]. Such challenges promoted the development of smart, targeted drug delivery systems capable of safely and precisely delivering anticancer drugs directly to tumor sites, resulting in enhanced therapeutic efficacy and minimizing systemic exposure [2]. These advanced delivery platforms provide several benefits, including improved pharmacokinetics, controlled drug release, decreased toxicity, and the capability to address multidrug resistance.

Resistance to chemotherapy remains a critical barrier to effective cancer treatment [6]. To overcome the limitations of conventional chemotherapeutic drugs, various strategies have been developed, including the use of liposomes and nanoparticles, which enhance drug efficacy and targeting capabilities [7]. Lipid and polymeric nanoparticles (LP-NCs), typically ranging in size from 1 to 1000 nm, encompass several types [8,9], such as liposomes (lipid vesicles), lipid nanoparticles, and polymeric nanoparticles. These nanocarriers can be loaded with anticancer drugs to improve drug accumulation and release at specific tumor sites, thereby maximizing therapeutic efficacy while minimizing systemic side effects [2]. They can also be engineered with a wide range of physicochemical and biological characteristics, such as sizes, shapes, surface charges, and polymer coatings, offering a multifunctional platform for disease detection and targeted therapy [8].

The use of LP-NCs offers several distinct advantages, including improved pharmacokinetic profiles of encapsulated drugs, prolonged circulation times, and passive targeting of tumors. This targeting is primarily facilitated by the phenomenon of the enhanced permeability and retention (EPR) effect, which promotes the accumulation and retention of liposomes and nanoparticles (typically 10 to 200 nm in size) due to impaired lymphatic drainage and increased vascular permeability at these sites [10]. Their nanoscale dimensions and tailored surface properties also help them evade rapid clearance by the reticuloendothelial system (RES), enhancing drug stability and biodistribution. Moreover, LP-NCs can reduce the systemic toxicity associated with unbound (free) drugs while increasing drug solubility and permeability, facilitating drug diffusion, and enabling the controlled and sustained release of encapsulated drugs [11]. In addition, they are generally biocompatible and, in certain cases, biodegradable, depending on carrier composition and structural design, and they exhibit minimal pharmacological adverse effects, as many are composed of lipids and polymers that are classified as “Generally Recognized as Safe (GRAS)” by the US Food and Drug Administration (US FDA) [12,13,14]. Collectively, these characteristics make LP-NCs a highly promising platform for advanced and targeted drug delivery applications. Despite these advantages, LP-NCs may still present potential toxicity or safety concerns depending on their composition, surface chemistry, and biodistribution profiles [15]. The use of LP-NCs enables controlled drug release and protects encapsulated drugs from rapid metabolism and systemic clearance. This ultimately improves patient compliance by reducing the frequency of dosing compared to conventional therapies [11]. Nevertheless, LP-NCs continue to face several persistent challenges. These challenges include their potential toxicity, *in vivo* accumulation, rapid clearance by the mononuclear phagocyte system, and difficulties in achieving consistent large-scale production with uniform physicochemical characteristics, which can be technically complex, financially demanding, and often hinder clinical translation [2,11]. Additionally, LP-NC stability remains a big concern due to formulation-related issues, such as self-aggregation and drug leakage. However, these challenges can be mitigated through lyophilization or freeze-drying techniques, which have shown considerable promise in improving the colloidal stability of LP-NCs by preventing aggregation, preserving particle size distribution, and extending shelf life during storage and transportation [16,17].

LP-NCs, despite their superior advantages and their potential to overcome prior limitations, often lack inherent specificity for tumor sites unless they are actively functionalized, which can result in off-target distribution and substantial adverse side effects in normal, noncancerous cells [10,18]. To overcome this key limitation and improve the targeting capabilities of chemotherapeutic drug delivery systems, photodynamic therapy (PDT) has emerged as a light-responsive, non-invasive therapeutic strategy that enables localized treatment at specific tumor sites [11]. PDT involves the local or systemic administration of photosensitizer (PS) molecules, which preferentially accumulate in specific tumor tissues and cells. Upon activation by light at a specific wavelength, these PS molecules generate reactive oxygen species (ROS), such as singlet oxygen (^1^O_2_), superoxide anions (O_2_^•−^), and hydroxyl radicals (^•^OH), leading to selective tumor cell damage while sparing surrounding healthy normal tissues [19,20]. A key advantage of PDT is that cytotoxic ROS are produced only upon light activation of the PS molecule, making the PDT highly selective and specific; in the absence of light, the PS molecule remains largely non-toxic [21]. Common PS molecules used in PDT include porphyrins (*e.g.*, Photofrin^®^, porfimer sodium; Pinnacle Biologics, Inc., Chicago, IL, USA), chlorins (*e.g.*, Chlorin e6, Ce6; Frontier Scientific, Inc., Logan, UT, USA), and phthalocyanines. However, these molecules often suffer from poor aqueous solubility, low tumor selectivity, and rapid systemic elimination, leading to limited therapeutic efficacy and undesirable side effects. On the other hand, PDT can be administered either before or after conventional treatments, such as chemotherapy, radiation, or surgery, without compromising their efficacy, offering flexibility in integrated cancer treatment options [22]. Compared with conventional cancer therapies, PDT offers enhanced targeting specificity and tumor selectivity, reduced side effects, and improved drug delivery efficiency [23]. Despite these advantages, the clinical translation of PDT faces big challenges, primarily due to the physicochemical and photophysical limitations of PS molecules. Most PS molecules are hydrophobic, which causes them to accumulate under normal physiological conditions, thereby reducing the efficiency of ROS generation [24]. Nanoscale drug delivery systems, particularly lipidic and polymeric nanocarriers, have been developed to efficiently encapsulate PS molecules, thereby enhancing their stability, bioavailability, and tumor accumulation *via* the EPR effect. When integrated into light-responsive nanocarrier systems, these platforms enable synchronized drug release and photodynamic activation, thereby improving treatment precision and minimizing off-target phototoxicity. Although recent advancements in PS molecule design and carrier systems have improved their properties, achieving selective accumulation at specific target sites remains inadequate for consistent therapeutic outcomes [25]. Consequently, the development of smart nanocarrier-based drug delivery systems capable of overcoming biological barriers is essential for maximizing the clinical potential of PDT. While numerous studies have explored the integration of nanocarriers with PDT, research specifically focused on light-responsive LP-NCs remains limited, emphasizing a significant gap for future investigation. Therefore, the aim of this study is to provide a comprehensive overview and meta-analysis of the development and application of light-responsive LP-NCs in PDT. This work emphasizes the critical role of photo-responsive moieties in cancer phototherapy and offers insights to guide future research and accelerate clinical translation. In particular, it demonstrates the value of bridging the gap between preclinical findings and clinical applications to achieve more effective and personalized cancer therapies.

## 2. Methods

### 2.1. Data Mining

Data was collected from databases, such as Embase^®^, Medline^®^, and PubMed^®^, as well as search engines, such as Scopus^®^, Google Scholar^®^, Web of Science^®^, and ScienceDirect^®^. The search utilized English-language keywords, including UV light, NIR light, smart, nanocarriers, lipid, polymeric, nanoparticles, liposomes, cancer therapy, photodynamic therapy (PDT), photoisomerization, photocleavage, photooxidation, photothermal, and photochemical.

**Figure 1:**
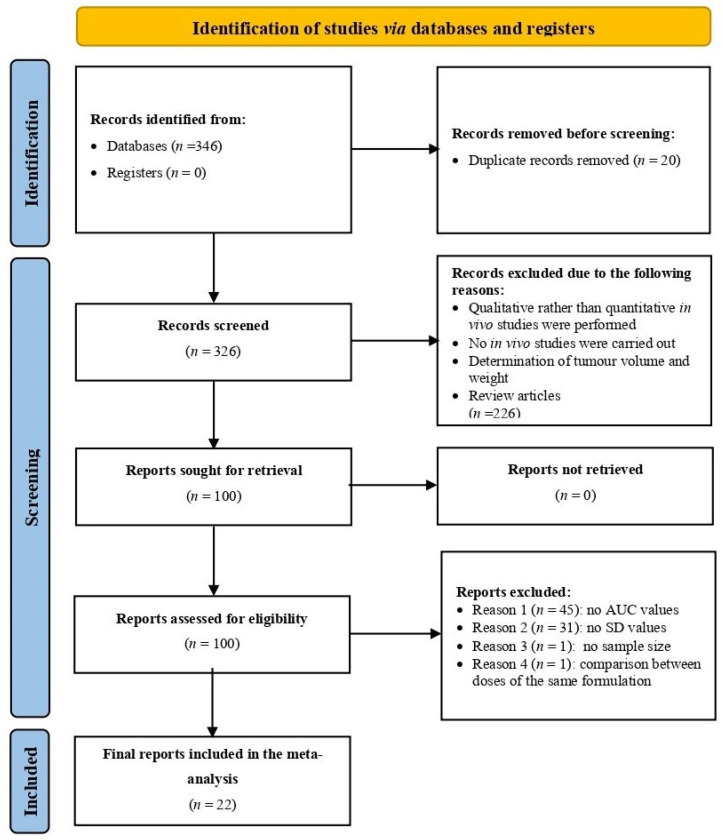
PRISMA flow diagram of the data mining process for light-responsive LP-NCs. Created in BioRender. Salazar, F. (2025) https://BioRender.com/d42tha7.

The literature search and data handling were processed in accordance with the PRISMA guidelines (Preferred Reporting Items for Systematic Reviews and Meta-Analyses), available at http://www.prisma-statement.org/ (accessed on 4 April 2025). Relevant studies were screened for duplicates, inclusion, and exclusion criteria, and data extraction was performed systematically. Figure 1 shows the entire data mining process in a flowchart diagram, providing a visual overview of study selection and data collection.

### 2.2. Inclusion Data and Criteria

The conducted meta-analysis relied on recording the area under the curve (AUC) as a key pharmacokinetic parameter. To be considered eligible for analysis, articles were required to meet the following criteria: (1) publication within the last ten years, (2) representation from diverse geographical locations, (3) use of different types of photoswitchable lipids, photoisomers, photosensitizers, and light wavelengths, (4) inclusion of a detailed methodology, (5) presentation of original data, and (6) a thorough explanation of “on-off” controlled photo-responsiveness in cancer therapy *in vivo*.

Although the initial screening covered a wide range of nanocarrier systems (including liposomes, lipid nanoparticles, polymeric nanoparticles, polymeric micelles, and inorganic nanocarriers), only studies involving lipidic or polymeric light-responsive nanocarrier systems reported sufficient quantitative pharmacokinetic data (AUC values) for inclusion. Other nanocarrier-based formulations or dosage forms did not meet the eligibility criteria or lacked comparable *in vivo* data, and were therefore excluded from the quantitative analysis. This selective inclusion ensured consistency and reliability in the meta-analysis, focusing on systems with robust pharmacokinetic characterization.

Following full-text evaluation, all eligible articles were rigorously reviewed and analyzed. Selected studies were required to contain original data and be published as research articles in recognized scientific databases. In addition, each article had to report the mean and standard deviation (SD) of the AUC, along with data from control groups treated with the studied drugs. Extracted data included the name of the anticancer drug, the number and type of animals used, the nanocarrier system, the photo-responsive moiety, the light source and wavelength, the authors’ names, and the publication year. The AUC was used as a pharmacokinetic indicator of bioavailability, allowing for comparison between drug-loaded light-responsive liposomes or nanoparticles and their respective controls. Table 1 summarizes the key characteristics of the studies included in this meta-analysis. Additional details on the light-triggered release mechanisms, as well as the specific light sources and wavelengths used, are summarized in Table S1.

### 2.3. Meta Analysis

An initial meta-analysis was conducted to assess the enhancement of anticancer drug bioavailability when encapsulated in light-responsive liposomes or nanoparticles, as demonstrated by the AUC, which served as the principal measure of effect. A meta-analysis collects results from several sources to provide a comprehensive and conclusive assessment. Accordingly, heterogeneity among the included studies was further calculated to evaluate the degree of variability.

OpenMetaAnalyst software version 12.11.14 (Released: 14 November 2012) (Brown University Center for Evidence-Based Medicine, Providence, RI, USA; http://www.cebm.brown.edu/openMeta/, accessed on 29 July 2025) was used to perform meta-analysis. For each study the standardized mean difference (SMD) and standard error (SE) were calculated using the reported AUC values, standard deviations (SD), and sample sizes (number of animals). Hedges’ g, a bias-corrected form of the SMD, was applied to adjust for small sample sizes [48]. The pooled SMD with its 95% confidence interval (CI) were calculated and presented as forest plots, the standard representation for this type of statistical analysis. A *p*-value of <0.05 was considered statistically significant.

The fundamental assumption of the fixed-effect model is that all studies estimate the same true effect, with differences arising only from random error. Nevertheless, this assumption was not applicable in the present study due to substantial variability among the included studies in the meta-analysis, which differed in drug formulations, tumor models, animal species, and administered doses. As a result, the overall effect size was estimated using a random-effects model, applying the DerSimonian–Laird (DL) method.

To further ensure the robustness of the results, the analysis was also repeated and validated using an alternative software package, Review Manager (RevMan) software version 5.4 (Cochrane Collaboration, Copenhagen, Denmark), which also employs the inverse variance method, but provides additional estimators for between-study variance, offering further validation of the overall effect size.

Heterogeneity was assessed using three key statistical parameters: the I^2^ index, the Q statistic, and the τ^2^, which represents between-study variance [49]. The I^2^ index quantifies the proportion of total variation across studies due to heterogeneity, while the Q statistic testes whether the observed variability in the effect size is greater than what could be expected by chance. The I^2^ index was calculated using the following Equation (1):

(1) $$I^{2}=max\;(0,\frac{Q-df}{Q}\times 100\%)$$

where df is the degree of freedom, calculated as the number of studies −1.

The τ^2^ was calculated using the random-effects model (DL method), as shown in the following Equation (2):

(2) $$\tau^{2}=\frac{Q-df}{C}$$

where C is a scaling factor used to calculate the τ^2^.

SMD was calculated using the following Equation (3):

(3) $$SMD=\frac{{Mean}_{a}-{Mean}_{b}}{S_{pooled}}$$

S_pooled_ was calculated using in the following Equation (4):

(4) $$S_{pooled}=\sqrt{\frac{\left(n_{a}-1\right)S_{a}^{2}+\left(n_{b}-1\right)S_{b}^{2}}{n_{a}+n_{b}-2}}$$

where S_a_ is the standard deviation (SD) of the mean effect of the light-responsive liposomes or nanoparticles, S_b_ is the SD of the mean effect of the conventional drug formulation (control), n_a_ is the number of animals exposed to the light-responsive liposomes or nanoparticles, and n_b_ is the number of animals receiving the conventional drug formulation (control).

Each study’s weight was determined using the inverse variance method, as shown in Equation (5):

(5) $$SW=\frac{1}{{SE}^{2}+\tau^{2}}$$

where SW refers to the weight of the study and SE to the standard error of the effect size. In this approach, studies with larger sample sizes and lower variability contribute greater weight, while studies with smaller sample size and higher variability contribute less.

The sensitivity and robustness of the meta-analysis were further validated using the leave-one-out method, in which one study is systematically removed at a time to assess the stability and consistency of the overall results.

## 3. Results

### 3.1. Overview of Included Studies

The meta-analysis was performed to assess whether light-responsive liposomes or nanoparticles provide superior bioavailability compared to conventional anticancer formulations. Bioavailability was evaluated using the AUC. A total number of 22 studies were identified through a systematic search and included in the meta-analysis. Given that multiple studies evaluated more than one liposomal- or nanoparticle-conventional comparison (*i.e.*, multiple arms per study), we initially conducted a preliminary meta-analysis that included all possible pairs for each study to assess the overall effect of the liposomal or nanoparticle formulation on the AUC. The results of the preliminary model showed a significantly greater pooled effect for the liposomal or nanoparticle formulation compared to the conventional formulation. Additionally, A leave-one-out sensitivity analysis showed that no single pair from any study affected the pooled effect size, as shown in Figures S1 and S2 and Tables S2 and S3 in the Supplementary Materials.

But, including more than one arm from the same study can result in overlapping effect estimates, which could violate the independence assumption needed for meta-analysis and potentially introducing bias into the pooled results. To maintain statistical validity and avoid over-representation of data from a single study, only a single comparison per study was considered, which was the pair with the lowest SMD to provide a conservative estimate and guarantee that each study contributed only once to the pooled analysis. Choosing the lowest SMD between the conventional formulation and the light-responsive liposome or nanoparticle formulation minimizes the risk of overestimation of the examined formulation over the conventional formulation and also ensures that the pooled estimate of the final meta-analysis reflects a conservative interpretation of the available evidence. This approach was aligned with the recommendations of the Cochrane Handbook, aimed at preventing unit-of-analysis errors and the artificial inflation of pooled estimates [48].

The included studies differed in drug formulations, tumor models, animal species, administered doses, and treatment durations. Sample sizes ranged from 3 to 8 animals per study group. In total, 92 animals were treated with light-responsive liposomes or nanoparticles, while another 92 animals received the conventional formulation.

### 3.2. Pooled Effect Size

**Figure 2:**
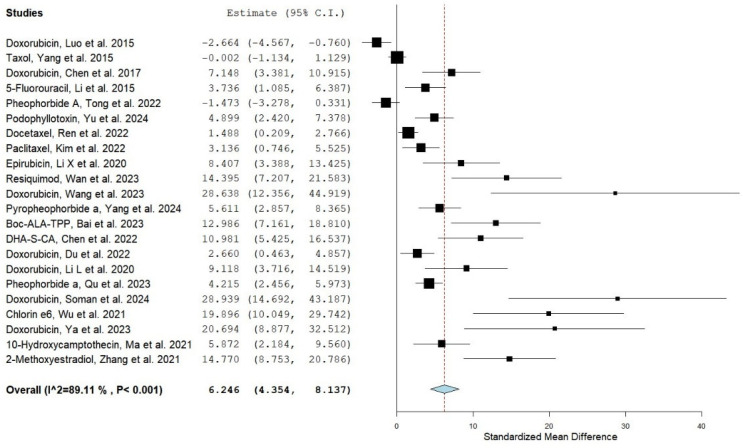
Forest plot presenting the preliminary meta-analysis results of the included studies [26,27,28,29,30,31,32,33,34,35,36,37,38,39,40,41,42,43,44,45,46,47], generated using OpenMetaAnalyst^®^ software version 12.11.14 (Released: 14 November 2012) (http://www.cebm.brown.edu/openMeta/, accessed on 29 July 2025). The black square represents the effect size of each study, and the horizontal line indicates the corresponding 95% confidence interval. The red dashed vertical line represents the overall pooled effect size of all included studies in the meta-analysis. The diamond at the bottom represents the pooled effect size and its 95% confidence interval, with the width of the diamond reflecting the precision of the overall estimate.

A random-effects model (DL method) was applied for the meta-analysis due to substantial variability among the included studies. The pooled effect size demonstrated a statistically significant improvement in bioavailability for the nanoencapsulation formulations compared with conventional formulations (SMD = 6.246, 95% CI: 4.354–8.137; *p* < 0.001). This indicates that, on average, liposomal or nanoparticle encapsulation of anticancer drugs markedly increases systemic exposure relative to conventional formulations. This finding is illustrated in Figure 2 (forest plot), which presents the effect sizes of individual studies alongside the overall pooled estimate. While the majority of studies consistently favored liposomal or nanoparticle-based drug delivery, some displayed wide confidence intervals overlapping zero, suggesting small or uncertain differences between the two tested formulations.

These results were consistent with the validation meta-analysis conducted using Review Manager (RevMan, version 5.4), which also demonstrated a significant pooled effect in favor of light-responsive liposomes and nanoparticles; the corresponding forest plot is presented in the Supplementary Materials (Figures S3 and S4).

### 3.3. Study Weights

The weights of the studies included are presented in Table 2 and were calculated using the inverse variance model, which accounts for both each study’s individual precision and the overall heterogeneity. The weights ranged from 1.11% (Wang *et al.*, 2023 [36]) to 6.24% (Yang *et al.*, 2015 [27]), indicating that no single study disproportionately influenced the overall findings. This approach ensures that studies with higher precision contribute more to the pooled estimates, while maintaining a balanced influence of all included studies on the final meta-analytic outcomes.

### 3.4. Heterogeneity of Effects

The heterogeneity analysis demonstrated substantial variability among the included studies (Q = 192.894, df = 21, *p* < 0.001; I^2^ = 89.113%, τ^2^ = 14.603), indicating considerable differences in effect sizes across studies. These results suggest that, although liposomal- or nanoparticle-based drug delivery systems generally enhance the bioavailability of anticancer agents compared with conventional formulations, the magnitude of this effect may vary depending on certain factors, such as the type of liposomal or nanoparticle formulation, the specific anticancer drug encapsulated, the animal model and tumor type, the administered dose, and the route of administration.

### 3.5. Sensitivity Analysis

**Figure 3:**
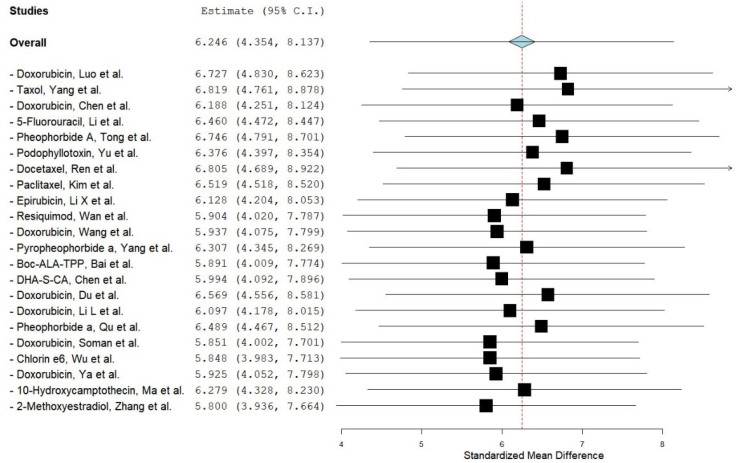
Leave-one-out sensitivity analysis illustrating the impact of sequentially removing each individual study on the overall effect size [26,27,28,29,30,31,32,33,34,35,36,37,38,39,40,41,42,43,44,45,46,47], generated using OpenMetaAnalyst^®^ software version 12.11.14 (Released: 14 November 2012) (http://www.cebm.brown.edu/openMeta/, accessed on 29 July 2025). The black squares represent the recalculated effect size obtained after removing each individual study, and the horizontal black lines indicate the corresponding 95% confidence intervals for these leave-one-out estimates. The red dashed vertical line represents the original pooled effect size calculated from all studies before any were removed. The diamond illustrates the overall pooled effect size and its 95% confidence interval when all studies are included.

A leave-one-out sensitivity analysis was performed to evaluate whether the pooled effect size was disproportionately influenced by any single study, as shown in Figure 3 and Table 3. The results demonstrated that sequential exclusion of individual studies did not materially alter the overall effect size, which remained statistically significant in all cases. This confirms the robustness of the findings and indicates that the observed superiority of liposomal- or nanoparticle-based drug delivery systems over conventional therapy was not driven by any single trial.

Table 3 summarizes the impact of excluding each study individually on the pooled effect size. The results show that the overall effect remained statistically significant across all iterations, confirming that no single study exerted an undue influence on the meta-analytic outcome.

### 3.6. Publication Bias

Egger’s regression test and the trim-and-fill method were applied to evaluate potential publication bias. Egger’s regression analysis yielded an intercept of 6.504 (*p* = 0.0001), indicating significant asymmetry in the distribution of effect sizes and suggesting the possibility of selective reporting in favor of positive findings. Consistent with this result, Duval and Tweedie’s trim-and-fill method estimated that approximately seven studies with potentially negative or neutral outcomes may be missing from the published literature.

After including these hypothetical studies, the adjusted pooled effect size under the random-effects model was SMD = 3.772 (95% CI: 1.873–5.672), indicating that the difference in AUC between the two formulations remained statistically significant. While the original pooled estimate (SMD = 6.246, 95% CI: 4.354–8.137) was higher than the adjusted estimate, suggesting that although publication bias may have slightly inflated the observed effect size, it does not eliminate the overall positive trend favoring liposomal and nanoparticle formulations over conventional drug delivery systems.

**Figure 4:**
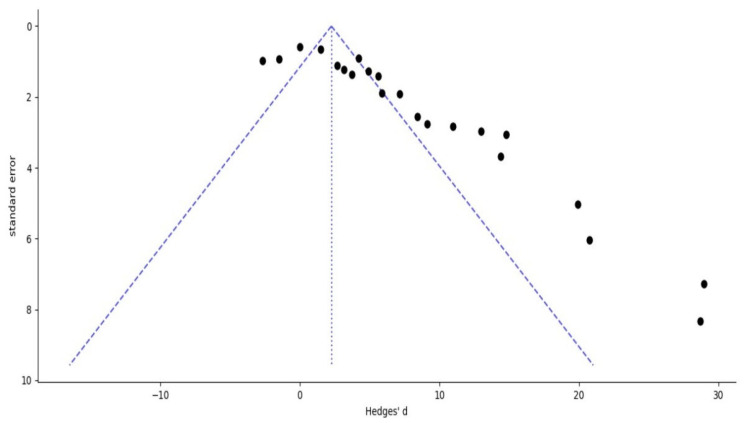
Funnel plot depicting publication bias among the studies included. Each dot represents a single study included in the meta-analysis, plotted according to its effect size (Hedges’ d, *x*-axis) and its precision (standard error, *y*-axis). The blue dashed lines represent the expected 95% confidence region of the funnel plot, while the blue vertical dotted line shows the overall pooled effect size. A symmetrical distribution of points suggests no publication bias, whereas an asymmetrical distribution may indicate possible bias.

Moreover, the funnel plot produced for this analysis showed marked asymmetry, with fewer studies located on the left side of the plot, visually supporting the statistical evidence of potential publication bias and indicating that smaller studies with negative or neutral results may be underreported (Figure 4).

## 4. Discussion

It is important to note that the present meta-analysis is limited to light-responsive lipid and polymeric nanocarriers, as no eligible studies using other nanocarrier-based formulations or dosage forms (such as polymeric micelles or inorganic nanoparticles) met the inclusion criteria with extractable pharmacokinetic data. Thus, the conclusions primarily reflect the efficacy of these two dominant nanocarrier systems currently supported by quantitative preclinical evidence.

Overall, this meta-analysis provides strong preclinical evidence that light-responsive liposomes and nanoparticles enhance the bioavailability of anticancer drugs compared with conventional chemotherapeutic formulations. These nanocarrier systems were associated with increased systemic exposure, prolonged circulation time, improved physical and chemical stability, and enhanced tumor targeting. While most studies favored nanoencapsulation over conventional formulations, the analysis revealed substantial heterogeneity and potential publication bias. The observed heterogeneity likely reflects differences in effectiveness across the included studies, which varied in nanoformulation (liposomal or nanoparticle-based, lipid or polymeric), the specific anticancer drug encapsulated, tumor type and animal model, administered dose, and treatment duration.

Publication bias, as indicated by the asymmetric funnel plot and confirmed by the trim-and-fill method, suggests the presence of missing studies, likely those with negative or neutral outcomes, which may have inflated the overall effect size. Nevertheless, the consistent direction of effect across most included studies favored nanoencapsulation, indicating that the trend toward improved bioavailability is robust, even if the magnitude of the benefit may be overestimated.

Given the limited number of preclinical trials investigating light-responsive liposomes and nanoparticles for specific cancer types, it was not feasible to conduct separate analyses for each cancer type. Accordingly, this meta-analysis synthesized all available evidence to provide an overarching evaluation of the potential advantages of light-responsive liposomal and nanoparticle formulations compared with conventional chemotherapeutic formulations. Beyond estimating a pooled effect, the review also aimed to map the existing research landscape, highlight methodological limitations, and inform priorities for future preclinical and clinical studies.

Furthermore, an initial exploratory meta-analysis was conducted to visualize the overall trend, including all 22 available treatment-control pairs from the included studies. This preliminary model showed a significant pooled effect in favor of the liposomal or nanoparticle formulations (SMD = 7.998; 95% CI: 6.429–9.568; *p* < 0.001), as shown in Figure S1 and Table S2, which was similar to the findings of the primary meta-analysis. This consistency across different analysis approaches reinforces the robustness of the findings and supports the overall reliability of the conclusion that light-responsive liposomes and nanoparticles are superior in terms of bioavailability than conventional formulations. Despite including non-independent data, the exploratory meta-analysis is considered illustrative rather than confirmatory. The alignment of its results with the primary analysis provides additional confidence in the observed trend.

Despite the heterogeneity observed across animal models and anticancer drug formulations, which contributed to variability in effect sizes, this aggregated analysis offers preliminary insights into the potential benefits of light-responsive liposomes and nanoparticles for cancer therapy. The findings highlight the need for adequately powered, standardized, and transparently reported animal studies, as well as more disease-specific investigations, to validate and translate these preclinical results into clinical applications.

Nevertheless, translating light-responsive liposomes and nanoparticles into clinical practice for the treatment of different types of cancer remains a challenge [50]. The key translational challenges include confirming safety profiles, optimizing pharmacokinetics, achieving large-scale manufacturing, and navigating complex regulatory regulations [50]. Comprehensive evaluation of biocompatibility, long-term toxicity, and biodistribution is crucial, as these nanocarriers may cause unpredictable systemic effects. In addition, hypersensitivity immune response can occur, especially in the case of PEGylated liposomes, where the immune reaction may involve hypothermia, hypotension, and impaired lung function [51]. Moreover, the intrinsic structural complexity of nanoparticles presents substantial obstacles to achieving scalable, reproducible manufacturing with consistent therapeutic and quality outcomes [52].

Regulatory frameworks for photo-responsive liposomes and nanoparticles are still evolving and require extensive data on preclinical and clinical settings, as well as rigorous quality control measures, which are challenging due to the current lack of standardized protocols for light-responsive nanocarriers [51]. To date, only a few conventional nanoformulations, such as liposomal-formulated doxorubicin (Doxil^®^, Janssen Pharmaceuticals, Horsham, PA, USA), PEGylated liposomal irinotecan (Onivyde^®^, Ipsen Biopharmaceuticals, Boulogne-Billancourt, France), and albumin-bound paclitaxel (Abraxane^®^, Bristol Myers Squibb, New York, NY, USA), have been approved by regulatory authorities (US FDA and European Medicines Agency (EMA)) and reached clinical use, mainly due to their robust characterization and reproducible production [51]. In turn, most light-responsive nanocarriers remain at the experimental stage. Future efforts should focus on standardized preclinical protocols, detailed toxicity assessments, and well-designed clinical trials to overcome translational challenges and unlock the full therapeutic potential of light-responsive nanocarriers in cancer therapy.

Nonetheless, several limitations warrant cautious interpretation. High heterogeneity and potential publication bias may have influenced the overall effect size. In addition, all included studies were conducted in animal models, and the selective inclusion of specific formulations to avoid statistical dependence may limit the generalizability of the findings. In addition, the small number of studies available for specific cancer types prevented subgroup analyses, rendering the present results exploratory rather than definitive.

## 5. Conclusions

This meta-analysis demonstrated that light-responsive liposomal and nanoparticle formulations of anticancer drugs improve bioavailability compared with conventional chemotherapeutic formulations. Enhanced bioavailability was associated with increased systemic exposure, prolonged circulation time, improved physical and chemical stability, and enhanced tumor targeting. Despite these promising results, the high heterogeneity and potential risk of publication bias across studies limit the certainty of the conclusions. These observations highlight the need for well-designed, adequately powered preclinical studies across different cancer types to validate these benefits and support the translation of this approach into clinical trials and eventual clinical application. Future research should prioritize the standardization of in vivo models and the transition toward early-phase clinical evaluation of light-responsive nanocarrier systems.

## Figures and Tables

**Table 1:** Summary of meta-analyses from published studies assessing the bioavailability of various nanocarrier systems for different anticancer drugs, compared with conventional delivery systems as controls.

No.	Drug/Study/Reference	Year	Group A Nanocarrier Drug Mean AUC (µg·h/mL)	Group B Conventional Drug Mean AUC (µg·h/mL)	Group A AUC-SD	Group B AUC-SD	Group A No. of Animals	Group B No. of Animals	SMD	UPPER CI	Lower CI	Type of Nanocarrier System
1	Doxorubicin/ Luo *et al.*, 2015 [26]	2015	415	1075	57	299	4	4	−2.664	−4.567	−0.76	HPPH-Liposomes
2	Taxol/ Yang *et al.*, 2015 [27]	2015	44.087	44.105	7.104	7.388	6	6	−0.002	−1.134	1.129	psCPP/NGR-NLC
3	Doxorubicin/ Chen *et al.*, 2017 [28]	2017	101.16	7.43	16.09	0.76	4	4	7.148	3.381	10.915	PCH-DI
4	5-Fuorouracil (ADR)/ Li *et al.*, 2015 [29]	2015	787.12	455.17	94.01	34.85	3	3	3.736	1.085	6.387	Irradiated Liposomes
5 *	Pheophorbide A (PA)/ Tong *et al.*, 2022 [30]	2022	241.24 *	257.9 *	11.47	5.59	3	3	−1.473	−3.278	0.331	cRGD–PaNPs–IgG
6 **	Podophyllotoxin (PPT)/ Yu *et al.*, 2024 [31]	2024	20.99 **	5.25 **	3.91	1.24	5	5	4.899	2.42	7.378	PPT LPs
7	Docetaxel (DTX/ Ren *et al.*, 2022 [32]	2022	0.00901694	0.00472472	0.00358859	0.00113927	6	6	1.488	0.209	2.766	DSD/HP NPs
8 *	Paclitaxel (PTX)/ Kim *et al.*, 2022 [33]	2022	6.01 *	0.77 *	1.86	0.31	3	3	3.136	0.746	5.525	EV(ICG/PTX)
9	Epirubicin (EPI)/ Li X *et al.*, 2020 [34]	2020	3.7713	0.7505	0.3569	0.1924	3	3	8.407	3.388	13.425	E/PCF-NPs
10	Resiquimod (RESQ)/ Wan *et al.*, 2023 [35]	2023	0.00002079	0.00000049	0.00000173	0.00000009	4	4	14.395	7.207	21.583	PCL8-TK-NA
11	Doxorubicin/ Wang *et al.*, 2023 [36]	2023	3.30021	1.42947	0.0566	0.04722	3	3	28.638	12.356	44.919	Fe_3_O_4_/DOX@CNSs
12 ***	pyropheophorbide a (PPa)/ Yang *et al.*, 2024 [37]	2024	13.007 ***	6.201 ***	1.324	0.803	5	5	5.611	2.857	8.365	HSSPAO Nas
13	BAT/ Bai *et al.*, 2023 [38]	2023	1331.684	703.43	58.034	21.135	5	5	12.986	7.161	18.81	BAT-NPs
14	(DHA-S-CA)/ Chen *et al.*, 2022 [39]	2022	22.90669	10.47263	1.22544	0.65818	4	4	10.981	5.425	16.537	IR808/DHA-S-CA NMs
15	Doxorubicin/ Du *et al.*, 2022 [40]	2022	61.625	26.485	12.34	8.36	3	3	2.66	0.463	4.857	Hemin/Dox-M
16	Doxorubicin/ Li L *et al.*, 2020 [41]	2020	74.54	25.55	5.532	2.481	3	3	9.118	3.716	14.519	Agpd@BSA/DOX
17	Pyropheophorbide-a (PA)/ Qu *et al.*, 2023 [42]	2023	262.8	93.26	53.38	6.51	8	8	4.215	2.456	5.973	PARE NP
18	Doxorubicin/ Soman *et al.*, 2024 [43]	2024	2.75523	0.99553	0.04504	0.05959	4	4	28.939	14.692	43.187	UIO-DOX
19	Chlorin e6 (Ce6)/ Wu *et al.*, 2021 [44]	2021	0.34151	0.1127	0.01261	0.00637	4	4	19.896	10.049	29.742	NCCe6/NCDTXL
20	Doxorubicin/ Ya *et al.*, 2023 [45]	2023	14.152	1.299333333	0.6945	0.09383333	3	3	20.694	8.877	32.512	CDLM
21	HCPT/ Ma *et al.*, 2021 [46]	2021	273.73	157.13	20.32	9.44	3	3	5.872	2.184	9.56	DPH NGs
22	2-ME/ Zhang *et al.*, 2021 [47]	2021	7.16666667	1.361666667	0.255	0.445	6	6	14.77	8.753	20.786	cRGDyk-2-ME@ICGP-TSL

**Notes:** * for study no. 5&8, the units are (% ID.h/mL); ** for study no. 6, the unit is (nmol·h/mL); and *** for study no. 12 unit is (µmol/L·h). **Abbreviations: BAT** (photosensitizer 5-aminolevulinic acid conjugated to the lipophilic cation triphenylphosphine (Boc-ALA-TPP)); **DHA-S-CA** (prodrugthioacetal, to bridge cinnamaldehyde (CA) and dihydroartemisinin (DHA)); **HCPT**(10-hydroxycamplothecin); 2-ME (2-methox-yestradiol); **HPPH-Liposomes** (2-[1-hexyloxyethyl]-2-devinyl pyropheophorbide-a lipososmes); **psCPP/NGR-NLC** (photo-sensitive cell penetrating peptides/Asn-Gly-Arg/nanostructured lipid carrier); **PCH-DI** (arylboronic ester and cholesterol modified hyaluronic acid (PPE-Chol1-HA)); **cRGD–PaNPs–IgG** (Cyclic arginine–glycine–aspartic acid/pheophorbide A nanoparticles/immunoglobulins); **PPT LPs** (Podophyllotoxin) liposomes; **DSD/HP NPs** (monosulfide-modified docetaxel (DTX) prodrugs (DSD)/hematoporphyrin (HP)) nanoparticles; **EV(ICG/PTX** (indocyanine green (ICG)- and paclitaxel (PTX)-loaded extracellular vesicles); **E/PCF-NPs** (epirubicin/lipid-PEG-cRGD (Cyclic arginine–glycine–aspartic acid) and lipid-PEG-Folic acid); **PCL8-TK-NA** (poly(ε-caprolactone)- thioketal-nanoagonist); **Fe_3_O_4_/DOX@CNSs** (caramelized nanospheres (CNSs) loaded with doxorubicin (DOX) and Fe_3_O_4_); **HSSPAO Nas** (1H,1H-hexylamine Oxygen carrying nanoassemblies); **BAT-NPs** (photosensitizer 5-aminolevulinic acid conjugated to the lipophilic cation triphenylphosphine (Boc-ALA-TPP)-nanoparticles); **IR808/DHA-S-CA NMs** (the near-infrared dye IR808 and the prodrug were adopted to prepare co-loaded Soluplus^®^/TPGS nanomicelles); **Hemin/Dox-M** (Hemin/doxorubicin dual-modal therapeutic nanoplatform); **Agpd@BSA/DOX** (Ag/Pd- bovine serum albumin-doxorubicin bimetallic nanozyme); **PARE NP** (Two small molecules, including a photosensitizer (pyropheophorbide-a, PA) and a Toll-like receptor 7/8 agonist (resiquimod, R848) conjugated into prodrug (PA-R848) that self-assembles into PA-R848 esterase responsive nanoparticles), **UIO-DOX** (UiO-66 metal–organic framework (MOF)-based system); **NCCe6/NCDTXL** (Nanocarrier chlorin e6 (Ce6) and nanocarrier docetaxel (Dtxl)); **CDLM** (Sonosensitizer chlorin e6 (Ce6) and chemotherapeutic agent doxorubicin (Dox) were co-loaded into microbubble-liposome complex to compose Ce6/Dox@Lip@MBs); DPH NGs (reductive cross-linking of purpurin 18 (P18) and 10- hydroxycamplothecin (HCPT) produces a well-designed prodrug nanogel (denoted as **DPH NGs**)); **cRGDyk-2-ME@ICGP-TSL** (photothermal therapeutic agent indocyanine green (ICG) and the chemotherapeutic drug 2-methoxyestradiol (2-ME), which were loaded into thermosensitive liposomes (TSLs) with surface-grafted tumor-targeting peptide cRGDyk.

**Table 2:** Relative weights of the studies included in the meta-analysis.

Drug Name	Study Author	Study Weight (%)
Doxorubicin	Luo *et al.*, 2015 [26]	5.99
Taxol	Yang *et al.*, 2015 [27]	6.24
Doxorubicin	Chen *et al.*, 2017 [28]	5.09
5-Fluorouracil	Li *et al.*, 2015 [29]	5.67
Pheophorbide A	Tong *et al.*, 2022 [30]	6.03
Podophyllotoxin	Yu *et al.*, 2024 [31]	5.75
Docetaxel	Ren *et al.*, 2022 [32]	6.20
Paclitaxel	Kim *et al.*, 2022 [33]	5.79
Epirubicin	Li X *et al.*, 2020 [34]	4.40
Resiquimod	Wan *et al.*, 2023 [35]	3.32
Doxorubicin	Wang *et al.*, 2023 [36]	1.11
Pyropheophorbide a	Yang *et al.*, 2024 [37]	5.62
Boc-ALA-TPP	Bai *et al.*, 2023 [38]	3.98
DHA-S-CA	Chen *et al.*, 2022 [39]	4.12
Doxorubicin	Du *et al.*, 2022 [40]	5.88
Doxorubicin,	Li L *et al.*, 2020 [41]	4.20
Pheophorbide a	Qu *et al.*, 2023 [42]	6.05
Doxorubicin	Soman *et al.*, 2024 [43]	1.38
Chlorin e6	Wu *et al.*, 2021 [44]	2.34
Doxorubicin	Ya *et al.*, 2023 [45]	1.83
10-Hydroxycamptothecin	Ma *et al.*, 2021 [46]	5.14
2-Methoxyestradiol	Zhang *et al.*, 2021 [47]	3.88

**Table 3:** Impact of excluding individual study on pooled effective size.

Drug/Study	Estimate	Lower Bound	Upper Bound	Std. Error	*p*-Value
Overall	6.246	4.354	8.137	0.965	<0.001
Doxorubicin/ Luo *et al.*, 2015 [26]	6.727	4.830	8.623	0.968	<0.001
Taxol/ Yang *et al.*, 2015 [27]	6.819	4.761	8.878	1.050	<0.001
Doxorubicin/ Chen *et al.*, 2017 [28]	6.188	4.251	8.124	0.988	<0.001
5-Fluorouracil/ Li *et al.*, 2015 [29]	6.460	4.472	8.447	1.014	<0.001
Pheophorbide A/ Tong *et al.*, 2022 [30]	6.746	4.791	8.701	0.998	<0.001
Podophyllotoxin/ Yu *et al.*, 2024 [31]	6.376	4.397	8.354	1.009	<0.001
Docetaxel/ Ren *et al.*, 2022 [32]	6.805	4.689	8.922	1.080	<0.001
Paclitaxel/ Kim *et al.*, 2022 [33]	6.519	4.518	8.520	1.021	<0.001
Epirubicin/ Li X *et al.*, 2020 [34]	6.128	4.204	8.053	0.982	<0.001
Resiquimod/ Wan *et al.*, 2023 [35]	5.904	4.020	7.787	0.961	<0.001
Doxorubicin/ Wang *et al.*, 2023 [36]	5.937	4.075	7.799	0.950	<0.001
Pyropheophorbide a/ Yang *et al.*, 2024 [37]	6.307	4.345	8.269	1.001	<0.001
Boc-ALA-TPP/ Bai *et al.*, 2023 [38]	5.891	4.009	7.774	0.961	<0.001
DHA-S-CA/ Chen *et al.*, 2022 [39]	5.994	4.092	7.896	0.970	<0.001
Doxorubicin/ Du *et al.*, 2022 [40]	6.569	4.556	8.581	1.027	<0.001
Doxorubicin/ Li L *et al.*, 2020 [41]	6.097	4.178	8.015	0.979	<0.001
Pheophorbide a/ Qu *et al.*, 2023 [42]	6.489	4.467	8.512	1.032	<0.001
Doxorubicin/ Soman *et al.*, 2024 [43]	5.851	4.002	7.701	0.944	<0.001
Chlorin e6/ Wu *et al.*, 2021 [44]	5.848	3.983	7.713	0.952	<0.001
Doxorubicin/ Ya et al., 2023 [45]	5.925	4.052	7.798	0.956	<0.001
10-Hydroxycamptothecin/ Ma et al., 2021 [46]	6.279	4.328	8.230	0.995	<0.001

## Data Availability

Not applicable.

## Supplementary Materials

The following supporting information can be downloaded at: https://www.mdpi.com/article/10.3390/ph18121796/s1, Table S1: Summary of Light Sources, Wavelengths, and Triggered Release Mechanisms; Figure S1: Forest plot of the exploratory meta-analysis including all liposomal and nanoparticle-conventional comparisons, generated using OpenMetaAnalyst® software (http://www.cebm.brown.edu/openMeta/, accessed on 29 July 2025); Table S2: Study weights for the exploratory meta-analysis including all liposomal and nanoparticle-conventional comparisons; Figure S2: Forest plot of the leave-one-out sensitivity analysis generated using OpenMetaAnalyst® software (http://www.cebm.brown.edu/openMeta/, accessed on 29 July 2025); Table S3: Results of the leave-one-out sensitivity analysis; Figure S3: Forest plot presenting the preliminary meta-analysis results of the included studies, generated using Review Manager (RevMan) software (version 5.4); Figure S4: Funnel plot depicting publication bias among the included studies, generated using Review Manager (RevMan) software (version 5.4).

## Abbreviations

^•^OH	Hydroxyl radicals
^1^O_2_	Singlet oxygen
AUC	Area under the curve
CI	Confidence interval
DL	DerSimonian–Laird
EMA	European Medicines Agency
EPR	Enhanced permeability and retention
GRAS	Generally Recognized as Safe
LP-NCs	Lipid and polymeric nanocarriers
NIR	Near Infrared
O_2_^•−^	Superoxide anions
PDT	Photodynamic therapy
PS	Photosensitizer
PRISMA	Preferred Reporting Items for Systematic Reviews and Meta-Analyses
RES	Reticuloendothelial system
RevMan	Review Manager
ROS	Reactive oxygen species
SD	Standard deviation
SE	Standard error
SMD	Standardized mean difference
US FDA	US Food and Drug Administration
UV	Ultraviolet
