# Supplementary material for: Advancing Photodynamic Cancer Therapy with Smart Light-Responsive Lipid and Polymeric Nanocarriers: Evidence from a Meta-Analysis of Efficacy and Pharmacokinetics
Source: Pharmaceuticals (Basel). 2025 Nov 25;18(12):1796. doi: 10.3390/ph18121796 (PMC12736384; doi:10.3390/ph18121796)
Supplement: Supplementary file 1 [file pharmaceuticals-18-01796-s001.zip › pharmaceuticals-3968327-supplementary.pdf]

Advancing Photodynamic Cancer Therapy with Smart Light-Responsive Lipid and Polymeric Nanocarriers: Evidence from a Meta-Analysis of Efficacy and Pharmacokinetics

Ahmed M. Agiba [1,*], Rabab A. El-Gazar [2], Mohamed A. Mekkawy [3], Nihal Elsayyad [4], Hala N. ElShagea [5], Patricia Segura-Medina [6,7,*] and Raghda Rabe Hamed [8]

Supplementary Materials

Table **1** in the manuscript summarizes the key characteristics of the studies included in this meta-analysis. Additional details regarding the light-triggered release mechanisms, along with the specific light sources and wavelengths employed across the included studies, are provided in Table **S1**.

Table S1. Summary of Light Sources, Wavelengths, and Triggered Release Mechanisms.

No.	Drug / Study	Photosensitizer	Light Source	Wavelength (nm)	Mechanism of Light-Triggered Release	Photodynamic Therapy Mechanism	References
1	Doxorubicin / Luo *et al.,* 2015	HPPH	NIR Laser	665 & 514	Micelle destabilization	Vascular targeted PDT causes tumor vasculature damage	[26]
2	Taxol / Yang *et al.,* 2015	psCPP	UV / Visible	365	Photoisomerization	ROS generation induces mitochondrial apoptosis	[27]
3	Doxorubicin / Chen *et al.,* 2017	NIR photothermal moiety	NIR Laser	808	Photothermal effect	ROS-enhanced apoptosis	[28]
4	5-Fluorouracil / Li *et al.,* 2015	PPDC	UV Light	365	Micelle destabilization	UV-triggered ROS apoptosis	[29]
5	Pheophorbide A / Tong *et al.,* 2022	PA	Red Diode Laser	660	Micelle destabilization	Type II PDT $^1O_2$ mediated immunogenic cell death	[30]
6	Podophyllotoxin / Yu *et al.,* 2024	ICG	NIR Laser	808	Photothermal effect	ROS cytotoxicity and chemo–PDT synergy	[31]
7	Docetaxel / Ren *et al.,* 2022	HP	Red Laser	630	Photocleavage	Type II PDT singlet oxygen-mediated apoptosis	[32]
8	Paclitaxel / Kim *et al.,* 2022	ICG	NIR Laser	808	Photothermal effect	ROS and photothermal therapy synergistic effects	[33]
9	Epirubicin / Li X *et al.,* 2020	PDA	NIR Laser	808	Photothermal effect	Hyperthermia-induced apoptosis with chemo synergy	[34]
10	Resiquimod / Wan *et al.,* 2023	PPA	NIR Light	808	Photocleavage	ROS-mediated tumor cell death and in situ vaccination	[35]
11	Doxorubicin / Wang *et al.,* 2023	Fe3O4@CNS	NIR Laser	808	Photothermal effect	Hyperthermia-enhanced chemotherapy	[36]
12	Pyropheophorbide a / Yang *et al.,* 2024	PPA	Red Laser	660	Micelle destabilization	Type II PDT singlet oxygen-mediated apoptosis	[37]
13	BAT / Bai *et al.,* 2023	ALA-TPP	Red/NIR Light	635	Micelle destabilization	ROS-mediated apoptosis aided by hypoxia relief	[38]
14	DHA-S-CA / Chen *et al.,* 2022	IR-808 dye	NIR Laser	808	Photothermal effect	ROS and photothermal chemo-cytotoxicity	[39]
15	Doxorubicin / Du *et al.,* 2022	Hemin (porphyrin)	Visible/NIR	630–660	Micelle destabilization	Type II PDT plus chemo synergy	[40]

16	Doxorubicin / Li L *et al.,* 2020	Plasmonic AgPd nanozyme	NIR Laser	808	Photothermal effect	ROS and hyperthermia combined to cause tumor cell death	[41]
17	Pyropheophorbide-a / Qu *et al.,* 2023	PPA	Red Laser	680	Nanoconjugate disassembly	Type II PDT and immune activation	[42]
18	Doxorubicin / Soman *et al.,* 2024	ICG	NIR Laser	808	Photothermal effect	Combined photothermal and ROS enhanced killing	[43]
19	Chlorin e6 / Wu *et al.,* 2021	Ce6	Red Laser	660	Micelle destabilization	Type II PDT singlet oxygen apoptosis	[44]
20	Doxorubicin / Ya *et al.,* 2023	Ce6	Visible Light & Focal Ultrasound	440	Micelle destabilization	ROS-mediated immunogenic cell death & checkpoint blockade	[45]
21	HCPT / Ma *et al.,* 2021	P18	Laser Beam	660	Photocleavage	Oxidative stress-induced apoptosis	[46]
22	2-ME / Zhang *et al.,* 2021	ICG	NIR Laser	808	Photothermal effect	ROS and photothermal therapy synergy	[47]

Abbreviations: ROS (reactive oxygen species); HPPH (porphyrin–phospholipid); **psCPP** (photo-sensitive cell pen-etrating peptides; **NIR** (Near infra-red); **ICG** (indocyanine green); **PPDC** (PID118-b-PLA71 diblock copolymer) UV-sensitive lipid component; **PA** (Pheophorbide A); **Ce6** (Chlorine6); **HP** (hematoporphyrin); **PDA** (Polydopamine)-photothermal material; **PPA** (Pyropheophorbide a)Photoactivatable nanoagonist; **Fe3O4@CNS** Magnetic carbon nanostructure; **ALA-TPP** (5-aminolevulinic acid conjugated with Triphenylphosphine) Mitochondria-targeted PS; **CA** (Cinnamaldehyde) ;**DHA** (dihy-droartemisinin); **IR808** (near-infrared dye); **GO** (Graphene Oxide); and **P18** (Purprin 18)-ROS-activatable prodrug.

Exploratory Meta-Analysis of All Treatment-Control Comparisons

An initial exploratory meta-analysis was conducted to visualize the overall trend, including all 22 available treatment-control pairs from the included studies. This preliminary model showed a significant pooled effect in favor of the liposomal or nanoparticle formulations (SMD = 7.998; 95% CI: 6.429-9.568; $p < 0.001$), as shown in Figure **S1** and Table **S2**.

A leave-one-out sensitivity analysis performed on this exploration model confirmed that the result was robust and not disproportionately influenced by any single comparison pair (Figure **S2** and Table **S3**).

This exploratory analysis includes non-independent data and is solely for illustrative purposes. The primary, methodologically robust analysis, which follows the single-comparison-per-study criterion, is elaborated in the main manuscript.

The exploratory and primary analyses were restricted to lipid and polymeric light-responsive nanocarriers. Other nanocarrier-based formulations or dosage forms identified in the broader literature search (*e.g.,* polymeric micelles and inorganic nanocarriers) lacked sufficient quantitative pharmacokinetic data (AUC values) for inclusion; therefore, these systems were excluded from the pooled analysis.

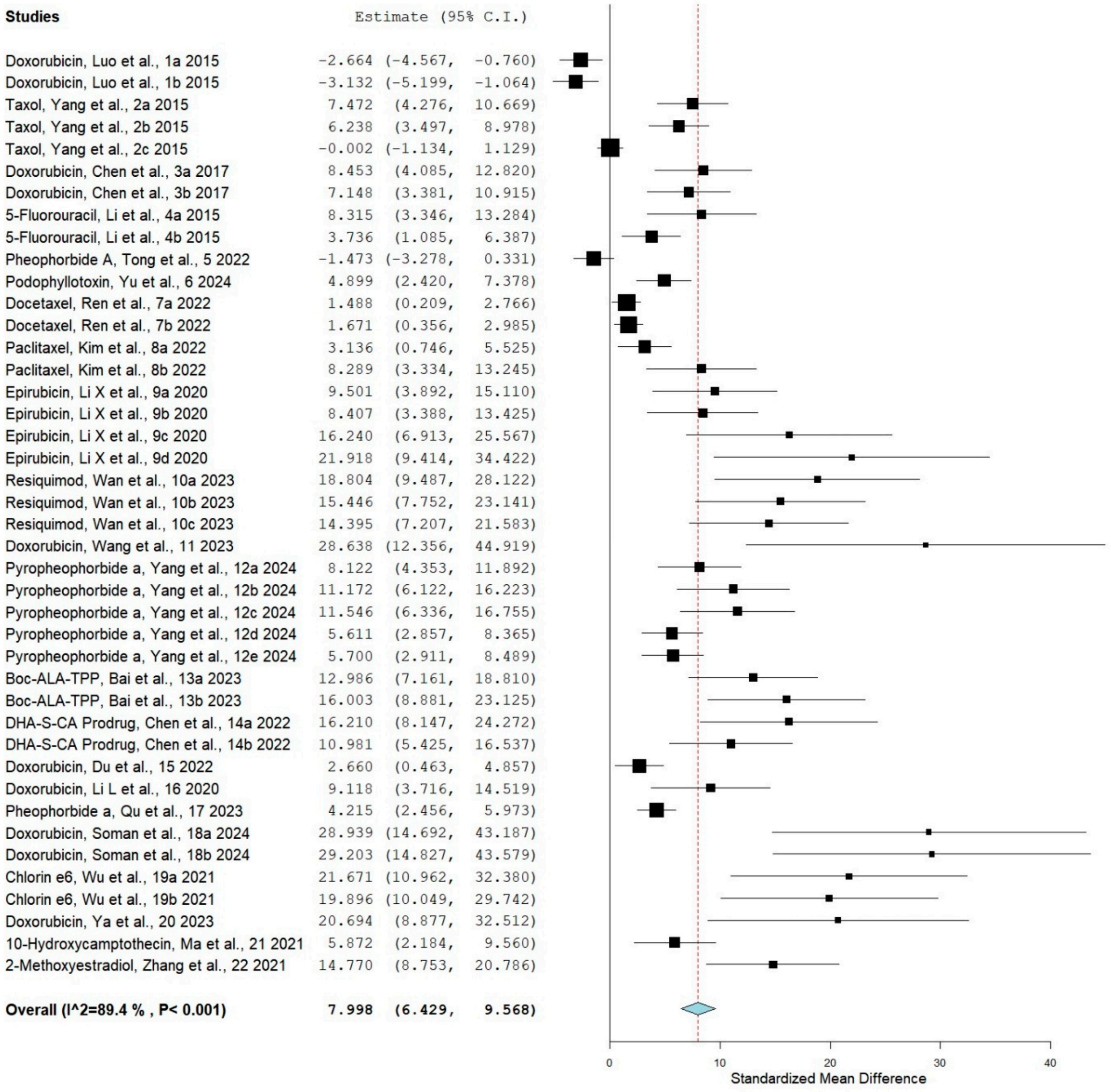

Figure S1. Forest plot of the exploratory meta-analysis including all liposomal and nanoparticle-conventional comparisons, generated using OpenMetaAnalyst® software (http://www.cebm.brown.edu/openMeta/, accessed 29 July 2025).

Table S2. Study weights for the exploratory meta-analysis including all liposomal and nanoparticle-conventional comparisons.

**Drug Name**	**Study Author**	**Study Weight (%)**
Doxorubicin (1a)	Luo *et al.,* 2015	3.178%
Doxorubicin (1b)	Luo *et al.,* 2015	3.151%
Taxol (2a)	Yang *et al.,* 2015	2.928%
Taxol (2b)	Yang *et al.,* 2015	3.026%
Taxol (2c)	Yang *et al.,* 2015	3.277%
Doxorubicin (3a)	Chen *et al.,* 2017	2.649%
Doxorubicin (3b)	Chen *et al.,* 2017	2.796%
5-Fluorouracil (4a)	Li *et al.,* 2015	2.498%
5-Fluorouracil (4b)	Li *et al.,* 2015	3.044%
Pheophorbide A (5)	Tong *et al.,* 2022	3.193%
Podophyllotoxin (6)	Yu *et al.,* 2024	3.077%
Docetaxel (7a)	Ren *et al.,* 2022	3.261%
Docetaxel (7b)	Ren *et al.,* 2022	3.257%
Paclitaxel (8a)	Kim *et al.,* 2022	3.094%
Paclitaxel (8b)	Kim *et al.,* 2022	2.502%
Epirubicin (9a)	Li X *et al.,* 2020	2.338%
Epirubicin (9b)	Li X *et al.,* 2020	2.486%
Epirubicin (9c)	Li X *et al.,* 2020	1.531%
Epirubicin (9d)	Li X *et al.,* 2020	1.070%
Resiquimod (10a)	Wan *et al.,* 2023	1.532%
Resiquimod (10b)	Wan *et al.,* 2023	1.850%
Resiquimod (10c)	Wan *et al.,* 2023	1.961%
Doxorubicin (11)	Wang *et al.,* 2023	0.726%
Pyropheophorbide a (12a)	Yang *et al.,* 2024	2.796%
Pyropheophorbide a (12b)	Yang *et al.,* 2024	2.478%
Pyropheophorbide a (12c)	Yang *et al.,* 2024	2.438%

Pyropheophorbide a (12d)	Yang *et al.,* 2024	3.023%
Pyropheophorbide a (12e)	Yang *et al.,* 2024	3.016%
Boc-ALA-TPP (13a)	Bai *et al.,* 2023	2.284%
Boc-ALA-TPP (13b)	Bai *et al.,* 2023	1.976%
DHA-S-CA Prodrug (14a)	Chen *et al.,* 2022	1.773%
DHA-S-CA Prodrug (14b)	Chen *et al.,* 2022	2.351%
Doxorubicin (15)	Du *et al.,* 2022	3.129%
Doxorubicin (16)	Li L *et al.,* 2020	2.390%
Pheophorbide a (17)	Qu *et al.,* 2023	3.199%
Doxorubicin (18a)	Soman *et al.,* 2024	0.889%
Doxorubicin (18b)	Soman *et al.,* 2024	0.878%
Chlorin e6 (19a)	Wu *et al.,* 2021	1.306%
Chlorin e6 (19b)	Wu *et al.,* 2021	1.441%
Doxorubicin (20)	Ya *et al.,* 2023	1.153%
10-Hydroxycamptothecin (21)	Ma *et al.,* 2021	2.815%
2-Methoxyestradiol (22)	Zhang *et al.,* 2021	2.237%

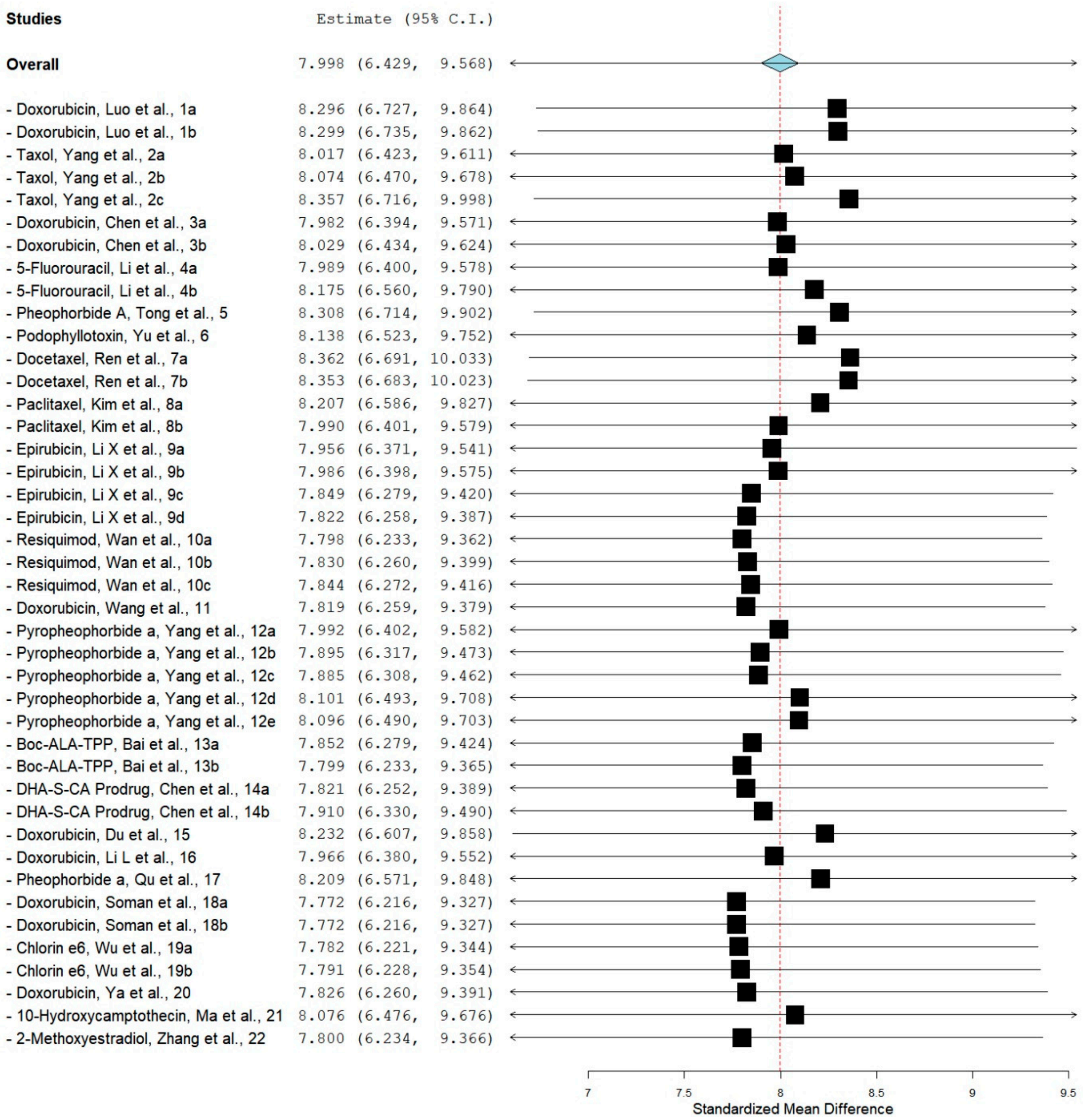

Figures S2. Forest plot of the leave-one-out sensitivity analysis generated using OpenMetaAnalyst® software (http://www.cebm.brown.edu/openMeta/, accessed 29 July 2025).

Table S3. Results of the leave-one-out sensitivity analysis.

Drug / Study	Estimate	Lower Bound	Upper Bound	Std. Error	*p*-Value
**Overall**	**7.998**	**6.429**	**9.568**	**0.801**	**<0.001**
Doxorubicin / Luo *et al.,* 2015 (1a)	8.296	6.727	9.864	0.800	<0.001
Doxorubicin / Luo *et al.,* 2015 (1b)	8.299	6.735	9.862	0.798	<0.001
Taxol / Yang *et al.,* 2015 (2a)	8.017	6.423	9.611	0.813	<0.001
Taxol / Yang *et al.,* 2015 (2b)	8.074	6.470	9.678	0.818	<0.001
Taxol / Yang *et al.,* 2015 (2c)	8.357	6.716	9.998	0.837	<0.001
Doxorubicin / Chen *et al.,* 2017 (3a)	7.982	6.394	9.571	0.811	<0.001
Doxorubicin / Chen *et al.,* 2017 (3b)	8.029	6.434	9.624	0.814	<0.001
5-Fluorouracil / Li *et al.,* 2015 (4a)	7.989	6.400	9.578	0.811	<0.001
5-Fluorouracil / Li *et al.,* 2015 (4b)	8.175	6.560	9.790	0.824	<0.001
Pheophorbide A / Tong *et al.,* 2022 (5)	8.308	6.714	9.902	0.813	<0.001
Podophyllotoxin / Yu *et al.,* 2024 (6)	8.138	6.523	9.752	0.824	<0.001
Docetaxel / Ren *et al.,* 2022 (7a)	8.362	6.691	10.033	0.853	<0.001
Docetaxel / Ren *et al.,* 2022 (7b)	8.353	6.683	10.023	0.852	<0.001
Paclitaxel / Kim *et al.,* 2022 (8a)	8.207	6.586	9.827	0.827	<0.001
Paclitaxel / Kim *et al.,* 2022 (8b)	7.990	6.401	9.579	0.811	<0.001

Epirubicin / Li X *et al.,* 2020 (9a)	7.956	6.371	9.541	0.809	<0.001
Epirubicin / Li X *et al.,* 2020 (9b)	7.986	6.398	9.575	0.810	<0.001
Epirubicin / Li X *et al.,* 2020 (9c)	7.849	6.279	9.420	0.801	<0.001
Epirubicin / Li X *et al.,* 2020 (9d)	7.822	6.258	9.387	0.798	<0.001
Resiquimod / Wan *et al.,* 2023 (10a)	7.798	6.233	9.362	0.798	<0.001
Resiquimod / Wan *et al.,* 2023 (10b)	7.830	6.260	9.399	0.801	<0.001
Resiquimod / Wan *et al.,* 2023 (10c)	7.844	6.272	9.416	0.802	<0.001
Doxorubicin / Wang *et al.,* 2023 (11)	7.819	6.259	9.379	0.796	<0.001
Pyropheophorbide a / Yang *et al.,* 2024 (12a)	7.992	6.402	9.582	0.811	<0.001
Pyropheophorbide a / Yang *et al.,* 2024 (12b)	7.895	6.317	9.473	0.805	<0.001
Pyropheophorbide a / Yang *et al.,* 2024 (12c)	7.885	6.308	9.462	0.804	<0.001
Pyropheophorbide a / Yang *et al.,* 2024 (12d)	8.101	6.493	9.708	0.820	<0.001
Pyropheophorbide a / Yang *et al.,* 2024 (12e)	8.096	6.490	9.703	0.820	<0.001
Boc-ALA-TPP / Bai *et al.,* 2023 (13a)	7.852	6.279	9.424	0.802	<0.001
Boc-ALA-TPP / Bai *et al.,* 2023 (13b)	7.799	6.233	9.365	0.799	<0.001
DHA-S-CA Prodrug / Chen *et al.,* 2022 (14a)	7.821	6.252	9.389	0.800	<0.001
DHA-S-CA Prodrug /	7.910	6.330	9.490	0.806	<0.001

Chen *et al.,* 2022 (14b)					
Doxorubicin / Du *et al.,* 2022 (15)	8.232	6.607	9.858	0.829	<0.001
Doxorubicin / Li L *et al.,* 2020 (16)	7.966	6.380	9.552	0.809	<0.001
Pheophorbide a / Qu et al., 2023 (17)	8.209	6.571	9.848	0.836	<0.001
Doxorubicin / Soman *et al.,* 2024 (18a)	7.772	6.216	9.327	0.794	<0.001
Doxorubicin / Soman *et al.,* 2024 (18b)	7.772	6.216	9.327	0.794	<0.001
Chlorin e6 / Wu *et al.,* 2021 (19a)	7.782	6.221	9.344	0.797	<0.001
Chlorin e6 / Wu *et al.,* 2021 (19b)	7.791	6.228	9.354	0.798	<0.001
Doxorubicin / Ya *et al.,* 2023 (20)	7.826	6.260	9.391	0.799	<0.001
10-Hydroxycamptothecin / Ma *et al.,* 2021 (21)	8.076	6.476	9.676	0.816	<0.001
2-Methoxyestradiol / Zhang *et al.,* 2021 (22)	7.800	6.234	9.366	0.799	<0.001

Results of meta-analysis using Review Manager (RevMan) software (version 5.4).

Validation meta-analysis performed using RevMan software (version 5.4) showed a significant pooled effect in favor of light-responsive liposomes and nanoparticles (SMD = 4.99, 95% CI: 3.15–6.84) (Figure **S3**). These results were consistent with the primary analysis conducted in Open Meta Analyst, confirming the increase in bioavailability with nanoformulations.

	Liposomal formula			Conventional formula				Std. Mean Difference	
Study or Subgroup	Mean	SD	Total	Mean	SD	Total	Weight	IV, Random, 95% CI	Year
Yang et al., 2015	44.087	7.104	6	44.105	7.388	6	7.8%	-0.00 [-1.13, 1.13]	2015
Li et al., 2015	787.12	94.01	3	455.17	34.85	3	5.8%	3.75 [-0.21, 7.70]	2015
Luo et al., 2015	415	57	4	1,075	299	4	7.1%	-2.67 [-4.97, -0.37]	2015
Chen et al., 2017	101.16	16.09	4	7.43	0.76	4	5.0%	7.16 [2.04, 12.27]	2017
Li X et al., 2020	3.7713	0.3569	3	0.7505	0.1924	3	3.1%	8.43 [0.13, 16.72]	2020
Li Y et al., 2020	74.54	5.532	3	25.55	2.481	3	2.8%	9.14 [0.17, 18.11]	2020
Ma et al., 2021	273.73	20.32	3	157.13	9.44	3	4.4%	5.89 [-0.02, 11.79]	2021
Wu et al.2021	0.34151	0.01261	4	0.1127	0.00637	4	1.5%	19.92 [6.15, 33.69]	2021
Zhang et al.2021	7.16666667	0.255	6	1.36166667	0.445	6	3.5%	14.78 [7.47, 22.08]	2021
Chen et al., 2022	22.90669	1.22544	4	10.47263	0.65818	4	3.3%	10.99 [3.31, 18.68]	2022
Ren et al., 2022	0.00901694	0.00358859	6	0.00472472	0.00113927	6	7.7%	1.49 [0.14, 2.83]	2022
Du et al., 2022	61.625	12.34	3	26.485	8.36	3	6.6%	2.67 [-0.36, 5.70]	2022
Kim et al., 2022	6.01	1.86	3	0.77	0.31	3	6.3%	3.14 [-0.29, 6.58]	2022
Tong et al.2022	241.24	11.47	3	257.9	5.59	3	7.2%	-1.48 [-3.62, 0.67]	2022
Wang et al.2023	3.30021	0.0566	3	1.42947	0.04722	3	0.4%	28.71 [0.94, 56.49]	2023
Ya et al.2023	14.152	0.6945	3	1.29933333	0.09383333	3	0.8%	20.75 [0.65, 40.85]	2023
Qu et al., 2023	262,800,000	53,380,000	8	93,260,000	6,510,000	8	7.3%	4.22 [2.27, 6.16]	2023
Wan et al., 2023	0.00002079	0.00000173	4	0.00000049	0.00000009	4	2.4%	14.41 [4.40, 24.42]	2023
Bai et al., 2023	1,331.684	58.034	5	703.43	21.135	5	3.5%	12.99 [5.57, 20.41]	2023
Yu et al.2024	20.99	3.91	5	5.25	1.24	5	6.6%	4.90 [1.88, 7.93]	2024
Soman et al., 2024	2.75523	0.04504	4	0.99553	0.05959	4	0.8%	28.97 [9.00, 48.94]	2024
Yang et al., 2024	13.007	1.324	5	6.201	0.803	5	6.3%	5.61 [2.22, 9.01]	2024
Total (95% CI)			92			92	100.0%	4.99 [3.15, 6.84]	

Heterogeneity: $Tau^2 = 11.03$; $Chi^2 = 114.39$, $df = 21$ ($P < 0.00001$); $I^2 = 82\%$

Test for overall effect: $Z = 5.32$ ($P < 0.00001$)

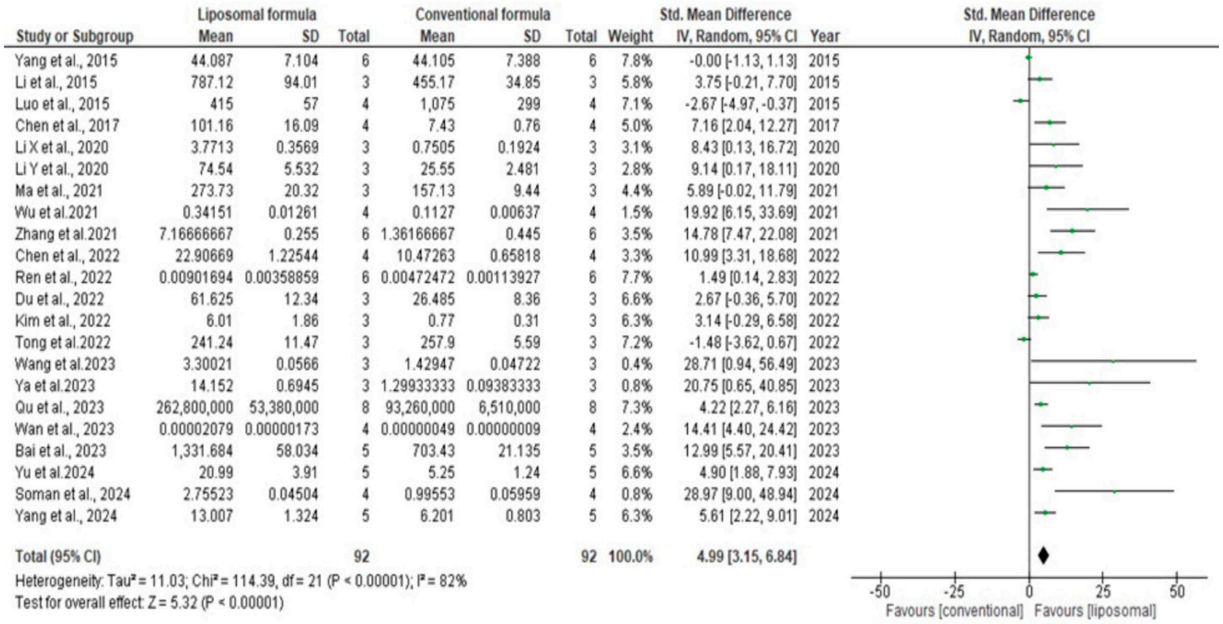

Figure S3. Forest plot presenting the preliminary meta-analysis results of the included studies, generated using Review Manager (RevMan) software (version 5.4).

The funnel plot generated using RevMan software (version 5.4) for validation of publication bias showed an asymmetric distribution of studies, further supporting the presence of potential bias, consistent with the primary analysis (Figure **S4**).

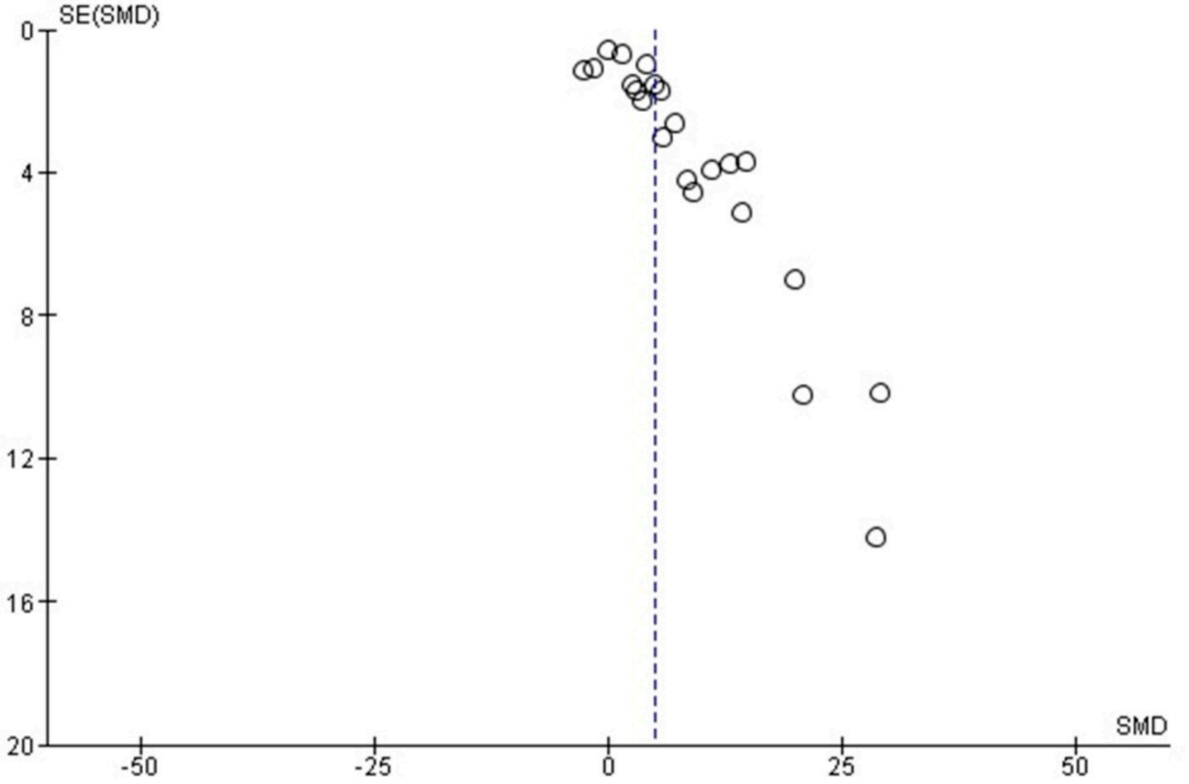

Figure S4. Funnel plot depicting publication bias among the included studies, generated using Review Manager (RevMan) software (version 5.4).
